# Unraveling the Developmental and Genetic Mechanisms Underpinning Floral Architecture in Proteaceae

**DOI:** 10.3389/fpls.2019.00018

**Published:** 2019-01-25

**Authors:** Catherine Damerval, Hélène Citerne, Natalia Conde e Silva, Yves Deveaux, Etienne Delannoy, Johann Joets, Franck Simonnet, Yannick Staedler, Jürg Schönenberger, Jennifer Yansouni, Martine Le Guilloux, Hervé Sauquet, Sophie Nadot

**Affiliations:** ^1^GQE-Le Moulon, INRA, Univ. Paris-Sud, CNRS, AgroParisTech, Université Paris-Saclay, Gif-sur-Yvette, France; ^2^Institute of Plant Sciences Paris-Saclay, CNRS, INRA, Universités Paris Diderot, Paris-Sud, Evry, Paris-Saclay, Gif-sur-Yvette, France; ^3^Ecologie Systématique Evolution, AgroParisTech, CNRS, Univ. Paris-Sud, Université Paris-Saclay, Orsay, France; ^4^Department of Botany and Biodiversity Research, University of Vienna, Vienna, Austria; ^5^National Herbarium of New South Wales (NSW), Royal Botanic Gardens and Domain Trust, Sydney, NSW, Australia

**Keywords:** Proteaceae, flower, development, floral symmetry, High Resolution X-Ray Computed Tomography, transcriptome, MADS-box genes, TCP genes

## Abstract

Proteaceae are a basal eudicot family with a highly conserved floral groundplan but which displays considerable variation in other aspects of floral and inflorescence morphology. Their morphological diversity and phylogenetic position make them good candidates for understanding the evolution of floral architecture, in particular the question of the homology of the undifferentiated perianth with the differentiated perianth of core eudicots, and the mechanisms underlying the repeated evolution of zygomorphy. In this paper, we combine a morphological approach to explore floral ontogenesis and a transcriptomic approach to access the genes involved in floral organ identity and development, focusing on *Grevillea juniperina*, a species from subfamily Grevilleoideae. We present developmental data for *Grevillea juniperina* and three additional species that differ in their floral symmetry using stereomicroscopy, SEM and High Resolution X-Ray Computed Tomography. We find that the adnation of stamens to tepals takes place at early developmental stages, and that the establishment of bilateral symmetry coincides with the asymmetrical growth of the single carpel. To set a framework for understanding the genetic basis of floral development in Proteaceae, we generated and annotated *de novo* a reference leaf/flower transcriptome from *Grevillea juniperina*. We found *Grevillea* homologs of all lineages of MADS-box genes involved in floral organ identity. Using *Arabidopsis thaliana* gene expression data as a reference, we found homologs of other genes involved in floral development in the transcriptome of *G. juniperina.* We also found at least 21 class I and class II TCP genes, a gene family involved in the regulation of growth processes, including floral symmetry. The expression patterns of a set of floral genes obtained from the transcriptome were characterized during floral development to assess their organ specificity and asymmetry of expression.

## Introduction

Proteaceae are a family of woody plants comprising approximately 1700 species in 81 genera, distributed mainly in the Southern Hemisphere, with two main centers of diversity, one in Australia and the other in South Africa. The hypogynous flowers almost invariably consist of four tepals (rarely 3 or 5), four stamens (rarely 3 or 5) opposite the tepals and with filaments that are adnate to the tepals (rarely free), and a single carpel with marginal placentation (Weston, 2007). Although this floral groundplan is highly conserved, the family displays considerable variation in other aspects of floral and inflorescence morphology. The inflorescence is basically a raceme but with various degrees of compaction. In subfamily Grevilleoideae, all but two genera are characterized by compound inflorescences consisting of racemes of flower pairs described as two-flowered short shoots sharing a common bract (Douglas and Tucker, 1996a). Early floral development has been described in detail in several species of Grevilleoideae, with emphasis on the ontogenic origin of the flower pair (Douglas and Tucker, 1996a) and carpel orientation (Douglas and Tucker, 1996b). In addition, Proteaceae have the highest number of transitions in perianth symmetry across all angiosperms (Reyes et al., 2016), with at least 10 transitions from actinomorphy (radial symmetry) to zygomorphy (bilateral symmetry) inferred throughout the family, and at least four reversals (Citerne et al., 2017). The developmental stage at which bilateral symmetry becomes visible varies across angiosperm species with a zygomorphic perianth; zygomorphy can be present from the very first stages, when the first floral organs are initiated, or can appear after all organs have been initiated and have begun to differentiate (Endress, 1999; Tucker, 1999). As in many taxa with a monomerous gynoecium, the single plicate carpel in Proteaceae becomes bilaterally symmetrical when the cleft starts to form, making the flower zygomorphic at the gynoecium level (Douglas and Tucker, 1996b; Sokoloff et al., 2017). Furthermore, in Proteaceae and more specifically in the subfamily Grevilleoideae, the high diversity of carpel orientation (dorso-ventral or oblique) adds further complexity when defining the orientation of the plane of symmetry relative to the common axis of the flower pair (Douglas and Tucker, 1996b).

The molecular bases of floral symmetry have been investigated in depth in *Antirrhinum majus* (Plantaginaceae). The *C**ycloidea* (*CYC*) gene is essential for the asymmetric development of the flower in the petal and stamen whorls (Luo et al., 1996, 1999). *CYC* belongs to a plant-specific transcription factor gene family, the TCP family, which is characterized by a non-canonical basic helix-loop-helix domain (the TCP domain, Cubas et al., 1999). TCP genes have been reported to be involved in various cell proliferation and growth processes that control organ development and shape (Huang and Irish, 2015; Nicolas and Cubas, 2016). In *A. majus*, *CYC* expression varies with the identity of the floral organ. Organ identity is classically determined by the combined action of four classes of genes, referred to as the A-, B-, C-, and E-classes (Coen and Meyerowitz, 1991; Causier et al., 2010). Petals are determined by the combined action of A- and B-class genes, stamens by B- and C-class genes, sepals by A-class genes only, and the carpel by C-class genes only. E-class genes act as obligate partners for all floral organ identity genes. The model is generally conserved across angiosperms with the notable exception of the A-class, whose function as defined in *Arabidopsis thaliana* appears to be limited to close relatives in the Brassicaceae. With the exception of one A function gene in *A. thaliana*, namely *A**petala**2*, these floral organ identity genes belong to the MADS-box transcription factor gene family (Theißen et al., 1996). B- and C-class genes may play a role in maintaining expression of *CYC* in the second and third floral whorls in *A. majus* (Clark and Coen, 2002).

Asymmetric expression patterns of *C**ycloidea* homologs (hereafter *CYC*-like genes) have been correlated with the independent evolution of zygomorphy in many monocot and eudicot clades (e. g., Hileman, 2014; Spencer and Kim, 2018). Functional studies have confirmed the role of *CYC*-like genes in the asymmetric development of floral organs in distantly related core eudicot species such as *Lotus japonicus*, *Iberis amara*, a hybrid of *Gerbera*, and in the monocot *Oryza sativa* (Feng et al., 2006; Busch and Zachgo, 2007; Broholm et al., 2008; Yuan et al., 2009). In orchids, however, no TCP gene has been found to date to be involved in zygomorphy. In this large monocot family, duplication and subfunctionalization in one of the two B-class gene lineages account for the inter- and intra-whorl tepal differentiation that generates zygomorphy (reviewed in Mondragon-Palomino, 2013). B-class genes have been found to be implicated in the independent evolution of zygomorphy in other monocot taxa. For instance, in *Commelina communis* (Commelinaceae), it has been suggested that in addition to the asymmetric expression of a *CYC*-like gene, zygomorphy is mediated by the asymmetric expression of a B-class gene in the inner tepal whorl associated with a change in ventral organ identity (from petal to sepal) (Preston and Hileman, 2012). In maize, the lack of expression of the B-class genes in the dorsal domain coincides with the absence of initiation of the dorsal lodicule (Bartlett et al., 2015).

Proteaceae, with their morphological diversity and their phylogenetic position within the basal eudicots, are good candidates for understanding the evolution of floral architecture, particularly regarding the homology of the undifferentiated perianth with either the calyx or the corolla of a differentiated perianth, and the repeated evolution of zygomorphy. In addition, the origin of the tetramerous perianth remains unclear. A recent study of floral trait evolution across angiosperms suggested that the ancestral perianth of eudicots may have been dimerous and undifferentiated (Sauquet et al., 2017), in which case the tetramerous perianth of Proteaceae could be of dimerous origin inherited from the ancestor of all eudicots. Yet, Proteaceae are relatively understudied from the point of view of developmental genetics. *CYC*-like genes have recently been characterized in Proteaceae. In two zygomorphic species of *Grevillea*, expression of the same *ProtCYC* gene was found to be asymmetric during the late stages of floral development (Citerne et al., 2017).

This paper combines developmental and transcriptomic data in *Grevillea juniperina* from subfamily Grevilleoideae. We compared early floral ontogenesis in *G. juniperina* with three other species presenting similar floral features but differing in floral symmetry, using a range of microscopy techniques (including High Resolution X-Ray Computed Tomography). We assembled *de novo* a transcriptome of *Grevillea juniperina* from RNA-seq of leaf and flower tissues. We searched this transcriptome for homologs of genes known to be involved in floral development in *Arabidopsis*, as well as focusing on the entire TCP and A-, B-, C-, E-class MADS-box gene families. We characterized the expression pattern during floral development of selected genes in *G. juniperina*.

## Materials and Methods

### Plant Material

**FIGURE 1:**
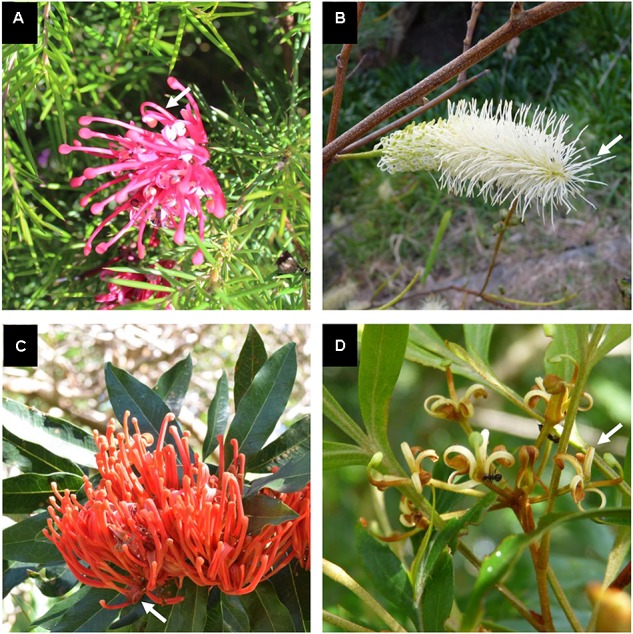
Inflorescences of the study species. All species have tetramerous flowers with stamens completely adnate to tepals. **(A)** Inflorescence of *Grevillea juniperina* with zygomorphic flowers at anthesis (arrow pointing to an open flower displaying curved tepals and style). **(B)** Inflorescence of *Grevillea petrophiloides* with actinomorphic flowers at anthesis (arrow pointing to an open flower displaying straight tepals and style). **(C)** Inflorescence of *Alloxylon flammeum* with zygomorphic flowers at anthesis (arrow pointing to an open flower displaying curved tepals and style). **(D)** Inflorescence of *Stenocarpus davallioides* with zygomorphic flowers at anthesis (arrow pointing to an open flower displaying curved tepals and style). Photographs S. Nadot **(A)** and H. Sauquet **(B–D)**.

Plant material (inflorescences at various pre-anthetic stages) was collected from four species of Proteaceae belonging to tribe Embothrieae of subfamily Grevilleoideae (Weston and Barker, 2006) and endemic to Australia: *Alloxylon flammeum* P. H. Weston & Crisp (subtribe Embothriinae), *Stenocarpus davallioides* D. Foreman & B. Hyland (subtribe Stenocarpinae), *Grevillea juniperina* R. Br. and *Grevillea petrophiloides* Meisn. (subtribe Hakeinae). The first three species have zygomorphic flowers while the latter has actinomorphic flowers (Figure 1). Detailed descriptions and illustrations of these species are available in the Flora of Australia online^1^. A dissection of the flower of *Grevillea juniperina*, showing all floral organs, is shown in Citerne et al. (2017).

Material for *Alloxylon flammeum* and *Stenocarpus davallioides* was sampled from trees cultivated at the Royal Botanic Garden Sydney (under accessions S2003-0047 and S1986-2016, respectively; voucher specimens were made (P.H.Weston 3425 and P.H.Weston 3426) and deposited at The National Herbarium of New South Wales (NSW)) and fixed in FAA (85 mL 55% ethanol, 5 mL glacial acetic acid, 10 mL formaldehyde). Material for *Grevillea petrophiloides* was sampled from trees cultivated in Longueville, NSW (voucher P.H.Weston 3582: High Resolution X-ray Computed Tomography) and Oakdale, NSW (voucher P.M.Olde 18/01: light microscopy) and fixed in FAA and 70% alcohol, respectively. Five plants of *Grevillea juniperina* were obtained from the garden center Truffaut and planted in the Parc Botanique de Launay (Orsay, France). These plants provided fresh material for the development analysis and for RNA extraction.

### Development Analysis

#### Light Microscopy and Scanning Electron Microscopy

Light microscopy was carried out on a Leica M125 stereomicroscope. For scanning electron microscopy, developing inflorescences and dissected tissues were dehydrated through an ethanol-acetone series, critical-point dried and sputter coated with gold. Imaging was carried out on a JEOL JSM 6390 Scanning Electron Microscope at 10 kV.

#### High Resolution X-Ray Computed Tomography (HRXCT) and 3D Reconstruction

For HRXCT, developing inflorescences of all four species were treated with a solution of 1% (w/v) phosphotungstic acid in FAA for 2 months (thereby ensuring saturation of the material with phosphotungstic acid), following the protocol of Staedler et al. (2013). The scans were performed on a MicroXCT-200 system (Zeiss Microscopy). X-Ray detection was performed via scintillator crystals (Zeiss Microscopy, in-house). The X-ray source was a Hammamatsu L9421-02 90 kV Microfocus X-Ray source. XMReconstructor 8.1.6599 (Zeiss Microscopy) was used to perform the 3D reconstruction from the scanning data. The AMIRA-based XM3DViewer 1.1.6 (XRadia Inc.) was used to visualize the scans. The reconstructed 3D data were then exported with XMController 8.1.6599 as a series of pictures in tiff format, typically ca. one to two thousand per sample. Scanning parameters are summarized in Supplementary Table S1.

### Tissue Collection and RNA Extraction

Young leaves and flower buds of *Grevillea juniperina* were collected and processed as in Citerne et al. (2017). Total RNA was extracted following a 2x CTAB-based extraction buffer and lithium chloride precipitation method (Smart and Roden, 2010) and total RNA was treated with DNase (Ambion) following the manufacturer’s instructions. For RNA-seq analysis, all tissues were harvested on the same plant: young leaves and floral buds of three size ranges (1–2 mm, 2–5 mm, over 6 mm long), and processed individually.

For qPCR analyses, floral buds from different plants were combined to constitute three biological replicates for flower dissections. Dissections were carried out on freshly harvested 6–7 mm buds already exhibiting bilateral symmetry under a stereomicroscope (6–10 buds from two–three plants for each biological replicate). Three tissue types (dorsal tepals with adnate stamens, ventral tepals with adnate stamens and gynoecium) were separately frozen in liquid nitrogen.

### Reference Transcriptome

#### Sequencing

Total RNAs of each tissue were checked for their integrity on a RNA_Nano chip, using an Agilent 2100 bioanalyzer (Agilent Technologies, Waldbronn, Germany), then pooled in equal amounts. The RNA-seq procedure was carried out at the Institute of Plant Sciences Paris-Saclay (IPS2, Saclay, France) on an IG-CNS Illumina Hiseq2000 platform. The library was constructed with the TruSeq stranded mRNA library Prep kit (Illumina^®^, CA, United States) with a sizing of 260 bp, and then sequenced in paired-end (PE) and a read length of 100 bases on a single lane. After quality trimming (removing the adapter, trimming of bases with a *Q* score <20 and removing pairs with at least one read <30 bases), 186,060,539 pairs of reads were generated. The raw data are available at SRA under the accession SRP141177.

#### Transcriptome Assembly

The *de novo* assembly of the transcriptome was performed following Roberts and Roalson (2017). Six assemblies were generated with Trinity v2.3.2 (Haas et al., 2013, with default parameters except –SS_lib_typeRF and –min_kmer_cov 3) and Velvet-Oases v1.2.09 (Schulz et al., 2012, kmers 25, 35, 45, 55, and 65) and combined with the tr2aacds.pl script (v 2014.05.15) of the Evidential Genes suite (Nakasugi et al., 2014). Only contigs classified as “main” were kept. The quality of the final assembly was assessed with BUSCO v3.0.2 (Waterhouse et al., 2018) using hmmer 3.1b2 against the embryophyta_odb9 dataset.

#### Homology Search and Functional Annotation

Predicted protein sequences from the *Grevillea juniperina* assembled transcriptome were annotated with BLASTP best hit (Camacho et al., 2009, *e*-value cutoff 1e-3) against a local version of the *Arabidopsis thaliana* protein database (TAIR 10). From BLASTP results, a gene ontology (GO) annotation was generated and extended through merging with InterProScan results using default parameters (Jones et al., 2014).

Overall homology of the *G. juniperina* proteome with other eudicot proteomes was assessed using OrthoVenn^2^ (Wang et al., 2015), selecting the proteomes of *Arabidopsis thaliana* and *Vitis vinifera*, and uploading the proteome of *Nelumbo nucifera* from NCBI (GCF_000365185.1_Chinese_Lotus_1.1_protein.faa.gz).

### Floral Gene Mining and Phylogenetic Analyses

#### Phylogeny of TCP Genes

All *Grevillea* contigs identified as TCP genes were extracted from the *G. juniperina* transcriptome annotated against the proteome of *Arabidopsis thaliana* by BLAST homology search. Sequence alignment was done with MUSCLE^3^ (Edgar, 2004) based on predicted amino acid translations, then manually refined. Phylogenetic reconstruction was done using maximum likelihood as implemented in PhyML (Guindon et al., 2010), using the GTR + Γ model [substitution model selected by AIC using the SMS option in PhyML (Lefort et al., 2017)] and the NNI method of tree optimization. Branch support was calculated with the aLRT SH-like method.

#### Phylogeny of ABCE MADS-Box Coding Sequences

In *Arabidopsis*, MADS-box genes with floral organ identity function are *A**petala**1* (*AP1*) (A-class), *A**petala**3* (*AP3*) and *P**istillata* (*PI*) (*B*-class), *A**gamous* (*AG*) (*C-*class) and *S**epallata* 1/2/4 (*SEP1/2/4*) and *S**epallata3* (*SEP3*) (*E-class*). We searched for homologs of these MADS-box genes in the *G. juniperina* transcriptome. Sixteen contigs were retrieved from the transcriptome following annotation against the *Arabidopsis thaliana* proteome. Sequences from transcriptomic data were translated, and seven full length protein sequences were used for subsequent analyses. MADS-box coding sequences (CDS) from *G. juniperina* were aligned with known floral MADS-box CDS of *Arabidopsis thaliana*^4^, *Nelumbo nucifera*^5^, *Aquilegia caerulea*^6^, and *Vitis vinifera*^6^, using MUSCLE based on predicted amino acid translations^7^ (protein IDs in Supplementary Table S2). Phylogenetic reconstruction was done using maximum likelihood as implemented in PhyML (Guindon et al., 2010), using the GTR + Γ + I model [substitution model selected by AIC using the SMS option in PhyML (Lefort et al., 2017)] and the NNI method of tree optimization. Branch support was calculated with the aLRT SH-like method.

#### Homologs of Floral Genes Co-expressed With ABCE MADS-Box Genes in *Grevillea juniperina*

We searched for the genes whose expression is correlated with that of the MADS-box genes implicated in the ABCE model in *A. thaliana*, excluding *SEP4* because of its low expression specificity to reproductive tissues. The search was done with the Expression Angler program using the AtGenExpress - Plus Extended tissue Compendium data set (Toufighi et al., 2005). The Pearson correlation coefficient (r-value) cut-off was fixed at 0.5 or 0.75, and a set of unique AGI (Arabidopsis Genome Initiative) gene identifiers (AGI-ID) was created. For each AGI-ID, the frequency of occurrence was calculated for the seven *A. thaliana* MADS-box genes used as baits and the best *r*-value reported (Supplementary Table S3). Then we searched for homologs of these genes in the *G. juniperina* transcriptome annotated against the *A. thaliana* proteome. A new dataset of the best matching AGI-ID in the *G. juniperina* transcriptome was obtained and the corresponding numbers of *G. juniperina* contigs were reported (Supplementary Table S3). A list of the 25 best correlated genes was also generated for each of the seven MADS-box genes used as baits and compared to the *G. juniperina* transcriptome.

### Quantitative RT-PCR Analyses of Floral Gene Expression

Thirteen *G. juniperina* contigs were selected based on their homology with genes expressed during floral development in *Arabidopsis thaliana*. They were renamed according to homology as follows: *A**gamous* (*GjuAG2*), *P**istillata* (*GjuPI2*), *S**epallata* (*GjuSEP1* and *GjuSEP3*), *A**petala**2* (*GjuAP2*), *A**petala**1* (*GjuAP1*), *W**uschel* (*GjuWUS*), *A**hootmeristemless* (*GjuSTM*), *C**up-shaped cotyledon**2* (*GjuCUC2*), *S**patula* (*GjuSPT*), *B**el1* (*GjuBEL1*), *T**ousled* (*GjuTSL*), and *C**rabsclaw* (*GjuCRC*). In addition, we included the *ProtCYC* genes (*GjuCYC1* and *GjuCYC2*) that were found to be expressed in flower buds by Citerne et al. (2017). Expression of these 15 genes was investigated in dissections of floral organs of pre-anthetic flowers (as described above).

Primer pairs were designed from the contig sequences (Supplementary Table S4). The *GjuPI2* gene corresponds to a contig closely related to the full length *GjuPI* used in the phylogenetic reconstruction, but lacking C-terminal region. Unfortunately, we were unable to define an appropriate pair of specific primers for *GjuAP1* from the available sequence. qRT-PCR reactions were performed using the Bio-Rad CFX384 touch (Bio-Rad, France) and the SYBR Premix Ex Taq (Tli RNaseH Plus) (Takara, Ozyme, France) following the supplier’s instructions. Three technical replicates were done for each of the three biological replicates of the floral dissections. A dilution series of the pooled cDNAs was used as a standard curve to validate the primer pairs and estimate the starting quantities (arbitrary units). Gene expression was normalized with the mean of the three reference genes *ACT8*, *TUB7*, and *FBA1* coding for actin, tubulin and fructose bisphosphate aldolase, respectively. The normalized expression values are provided in Supplementary Table S5. Differential expression for each gene was tested with a Two-way ANOVA (tissue and replicate effects) and a Tukey test for pairwise comparisons. *P*-values were adjusted for multiple testing with a BH correction. Adjusted *p*-values lower than 0.05 were considered as significant.

## Results

### Floral Development and Establishment of Zygomorphy

**FIGURE 2:**
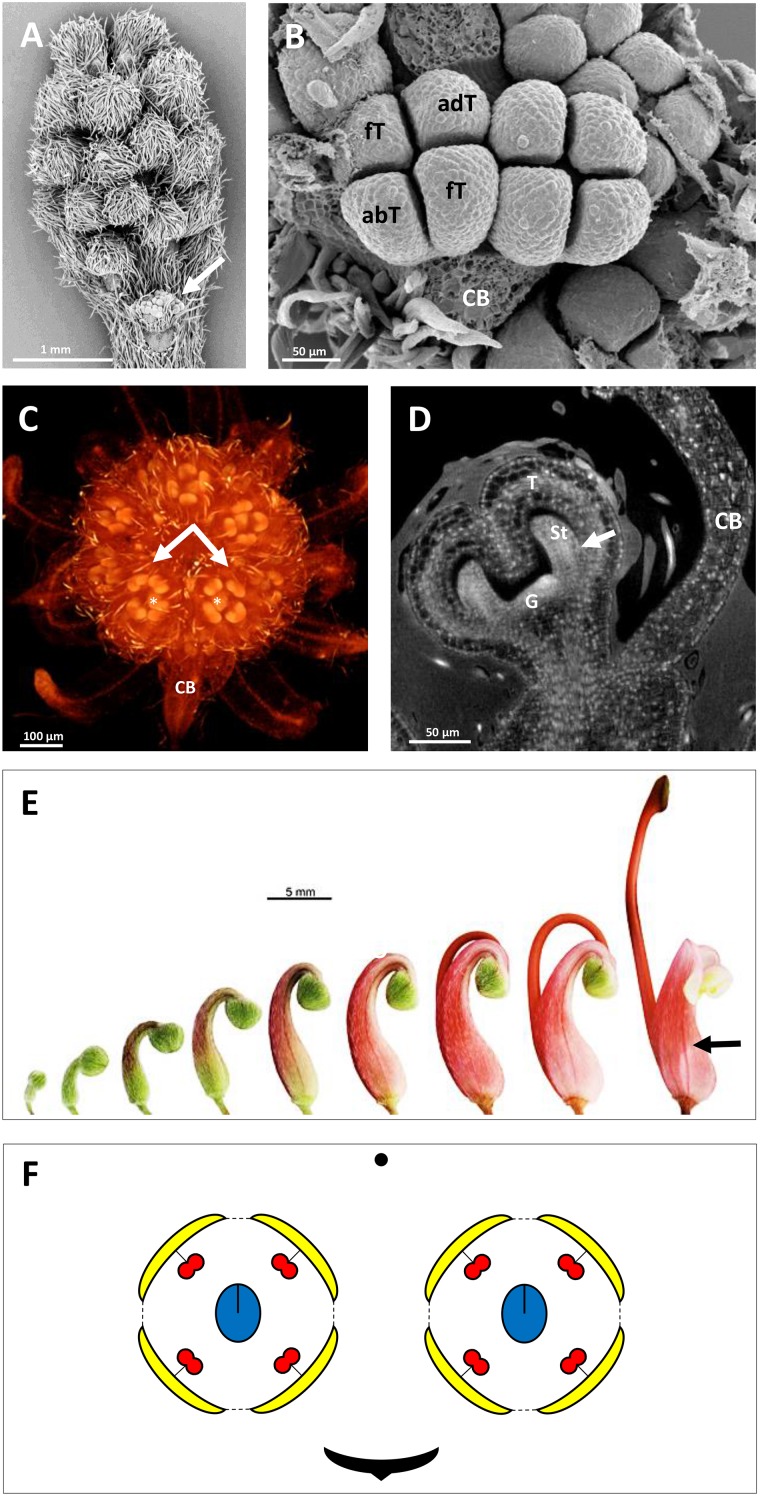
Inflorescence and flower pair development in *Grevillea juniperina* (**A,B**: SEM; **C,D**: CT-scans; **E**: stereomicroscope). **(A)** Whole inflorescence, composed of a first order axis bearing secondary order axes each surrounded by a hairy subtending bract. Each secondary axis bears pairs of flowers (conflorescences). The arrow points to one secondary order axis (subtending bract removed). **(B)** Flower pair, with common subtending bract (CB) removed, showing incomplete valvate aestivation of the tepals (fT, frontal tepal; adT, adaxial tepal = dorsal tepal; abT, abaxial Tepal = ventral tepal). **(C)** Top view of a second order axis of inflorescence, showing a flower pair from top view (arrows) with all organs initiated; the flower is made zygomorphic by the non-central position of the gynoecium (asterisks). **(D)** Virtual longitudinal section of a young flower bud showing the monosymmetrical gynoecium primodium (G); the arrow points to the fusion between a stamen and a tepal. **(E)** Floral developmental sequence, from the first stage at which zygomorphy becomes conspicuous (left) to anthetic flower. The arrow points to the strongly zygomorphic ovary visible through the perianth. **(F)** Floral diagram of a flower pair of *Grevillea juniperina* showing the orientation of each flower relatively to the common bract.

**FIGURE 3:**
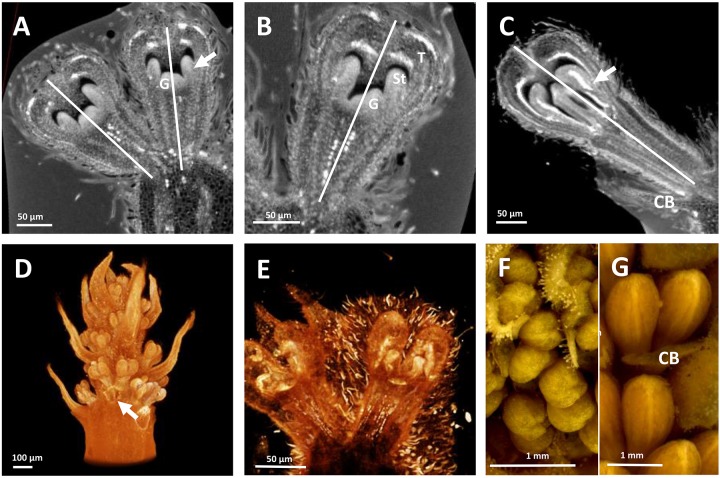
HRXCT images of developing flowers and flower pairs in *Stenocarpus davallioides* **(A–C)**, *Alloxylon flammeum* **(D,E)** and HRXCT and stereomicroscope images of inflorescence and flower pairs in *Grevillea petrophiloides* **(F,G)**. **(A)** Virtual longitudinal section of a flower pair (lateral view), with slight monosymmetry of the gynoecium (G) with regard to the vertical floral axis, visible on the right; the arrow points to the fusion between a stamen and a tepal. **(B)** Virtual longitudinal section of a flower (front view), showing the monosymmetry of the gynoecium in the vertical axis, while the tepals (T) and adnate stamens (St) are identical. **(C)** Virtual longitudinal section of a flower at a later stage (lateral view), showing slight asymmetry of the tepals and adnate stamens (the arrow points to the fused zone between a stamen and a tepal) with respect to the vertical axis (CB, common bract). **(D)** Lateral view of a whole inflorescence, with the common subtending bract of a flower pair removed (arrow). **(E)** Lateral view of a flower, zygomorphy is not yet apparent. **(F,G)** Lateral view of flower pairs at two developmental stages (CB, common bract).

The developing flowers in *Grevillea juniperina* (zygomorphic flowers at anthesis, Figure 1A, arrow) display a perianth that is actinomorphic until the tepals are formed and begin to elongate (Figures 2A–F), as in the actinomorphic species *Grevillea petrophiloides* (Figure 1B, distal part of the inflorescence, and Figures 3F,G). In *G. juniperina*, the virtual longitudinal section obtained with HRXCT shows that the carpel develops asymmetrically relative to the center of the floral bud (Figure 2D), even though the other organ whorls (tepals and stamens) are radially symmetrical. The monosymmetrical shape of the developing carpel suggests asymmetrical growth of the primordium. It can be noted that the incomplete valvate aestivation in *G. juniperina* (Figure 2B) makes the young flower bud slightly disymmetric rather than strictly actinomorphic. The base of the tepals and stamens is already slightly fused, indicating that the adnation of stamens to tepals occurs early during development. Early development is similar in the two other species sampled in this study, *Alloxylon flammeum* and *Stenocarpus davallioides* (both zygomorphic at anthesis; Figures 1C,D, arrows), which have flowers with an actinomorphic perianth at early stages and a gynoecium that develops asymmetrically relative to the center of the floral bud (Figures 3A–E). In *Grevillea petrophiloides*, the style elongates faster than the tepals before anthesis, and forces its way through the two dorsal tepals (visible in the proximal part of the inflorescence, Figure 1B), but in mature flowers, the style and the tepals are straight, contrary to the three other species.

### Assembly and Annotation of the Reference Transcriptome

67,004 contigs were obtained from the *de novo* assembly of the transcriptome of *G. juniperina*, with lengths ranging from 180 to 9,937 bases for a mean of 877.8, a N50 of 1,491 and 59,306,330 assembled bases. The result of the BUSCO analysis was C: 84.8% [S: 74.9%, D: 9.9%], F: 8.4%, M: 6.8%, n: 1,440 demonstrating the good quality of this assembly.

InterProScan identified 30,063 contigs with at least one protein domain. Among these, 14,488 contigs could be annotated with one category (or more) of the Gene Ontology (GO). We focused on transcription factors, selecting all contigs annotated as GO:0003700 and analyzed their PFAM domains. We found 240 contigs with such transcription domain annotation (1.6% of the GO annotated contigs), most of which corresponded to AP2 (29%), WRKY DNA-binding (23%) and bZip (12%) domains (Supplementary Figure S1).

37,842 contigs had homology with 13,946 *Arabidopsis* proteins, while 37,817 had a GO annotation. The two best-represented categories in Biological Process were “other cellular processes” (26%) and “other metabolic processes” (22%); in Cellular Components, the four best-represented categories were “other cytoplasmic components” (18%), “other intracellular processes” (14%), “nucleus” (15%) and “other membranes” (13%); among Molecular Functions, the prominent categories were “protein binding” (20%), “other binding” (14%),“other enzyme activity” (13%), and “transferase activity” (11%) (Supplementary Figure S2). We found 937 *G. juniperina* contigs with a functional annotation in one or more categories identified as GO: 0009908 (flower development) and its children categories. Beyond general flower development, the most represented categories pertained to stamen (20%) and ovule and carpel (16%) development (Supplementary Figure S3).

### Homology With Other Eudicot Proteomes

**FIGURE 4:**
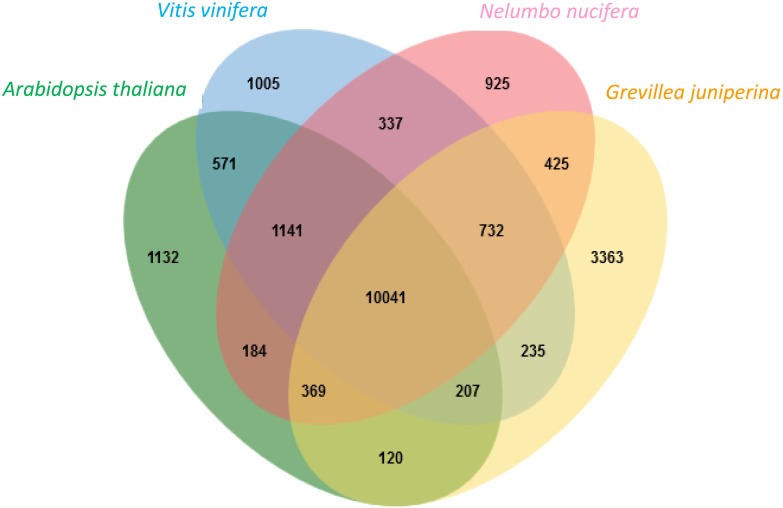
Distribution of clusters (orthologous groups) between the *Arabidopsis thaliana*, *Vitis vinifera*, *Nelumbo nucifera* proteomes and the *Grevillea juniperina* leaf/flower proteome.

Proteins deduced from the contigs of *G. juniperina* were compared to those in the proteomes of three fully sequenced species, *Arabidopsis thaliana*, *Nelumbo nucifera*, and *Vitis vinifera*, using to the algorithm available from the OrthoVenn site (Figure 4). As expected because of imperfect transcriptome *de novo* assembly, the highest proportion of singletons was obtained from *G. juniperina* (58% of the number of proteins against 10–33% in the three other species). 20,787 clusters, i.e., orthologous groups were inferred, 14,362 of these included at least two species. 3,363 clusters included only sequences from *G. juniperina* while 1,132, 1,005, and 925 clusters were specific to *A. thaliana*, *V. vinifera*, and *N. nucifera*, respectively. This higher number of specific clusters could be due to various factors (distant homologs that were not identified, enrichment in specific floral symmetry genes, assembly problems, etc). A GO enrichment test on these cluster members did not provide any clues as to a possible involvement in floral symmetry. 10,041 orthologous groups included sequences from all four species. Among the three pairwise comparisons involving the inferred *Grevillea* proteome, *Grevillea* and *Nelumbo*, both from the order Proteales, shared the highest number of total and pair-specific orthologous groups, which is consistent with their phylogenetic position. Orthologous groups including sequences of *Grevillea*, *Vitis*, and *Nelumbo* were more numerous than those including sequences of *Arabidopsis*, *Vitis*, and *Nelumbo*.

### Mining the *Grevillea juniperina* Transcriptome for Homologs of Genes Involved in Floral Development

We focused on two families of transcription factors involved in growth and development processes, particularly in flowers. TCP genes have been reported to be involved in various cell proliferation and growth processes that control organ development and shape, including floral symmetry (Huang and Irish, 2015; Nicolas and Cubas, 2016). MADS-box genes of A-, B-, C-, and E- classes are involved in floral organ identity and also interact with patterning and growth genes (Sablowski, 2015). In addition, we searched for homologs of genes co-expressed with the ABCE MADS-box genes as candidates for a conserved floral gene regulatory network module between *A. thaliana* and *G. juniperina*.

#### Phylogeny of TCP Homologous Genes

Twenty-four contigs with homology to TCP genes were identified in our *G. juniperina* transcriptome. Assembled fragment lengths ranged from 245 to 4,726 nucleotides; 10 fragments were predicted to contain complete ORFs (with identifiable start and stop codons) with putative ORF lengths ranging from 211 to 573 amino acids. All contigs but three had the characteristic basic helix-loop-helix TCP domain. Of the three contigs where the TCP domain could not be found, two had 76.4% sequence identity with R domains that were identified by blastx searches as homologous to TCP2. One contig lacked any recognizable domain because it corresponded to the region downstream of the TCP domain, but was found to be homologous by blastx searches to TCP8.

**FIGURE 5:**
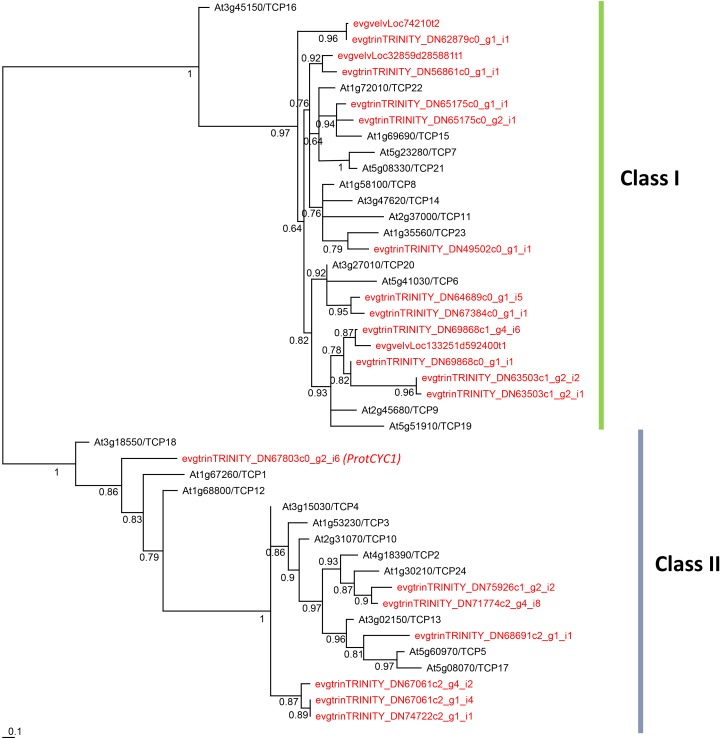
Unrooted phylogram of TCP genes from the *Arabidopsis thaliana* genome and the *Grevillea juniperina* transcriptome (in red). 21 of the 24 *G. juniperina* transcripts were included in this analysis. Branches with less than 0.5 aLRT values were collapsed. Representatives of both class I and class II TCP genes were found in *G. juniperina*.

The phylogenetic analysis of 192 aligned nucleotide positions, mainly comprising the TCP domain, from the 21 *G. juniperina* (excluding the three contigs without TCP domain) and 24 *A. thaliana* TCP genes showed that both class I and class II TCP were recovered with high support (Figure 5). Class I genes were over-represented in *G. juniperina*, with at least 14 copies compared to *A. thaliana* (13 copies), unlike class II genes with at least seven copies compared to *A. thaliana* (11 copies). One *CYC*-like copy (homologous to the *CYC*-like genes TCP18/TCP12/TCP1 in *A. thaliana*) was found in the transcriptome of *G. juniperina*, corresponding to the previously characterized *ProtCYC1* (Citerne et al., 2017).

#### Phylogeny of ABCE MADS-Box Genes

Sixteen contigs annotated as one of the *Arabidopsis thaliana* ABCE MADS-box AGI gene identifiers were extracted from the *G. juniperina* transcriptome after annotation. Seven fragments were predicted to contain full length ORFs (with identifiable start and stop codons) with putative lengths ranging from 209 to 247 amino acids. All full-length contigs had the characteristic MIKC domains. Among the nine incomplete contigs, four lacked the N-terminal coding sequence and five lacked the C-terminal region. Among these incomplete sequences, one, three, and five contigs aligned partially with *GjuAP1*, *GjuSEP*, and *GjuPI*, respectively, suggesting other alleles or paralogs.

**FIGURE 6:**
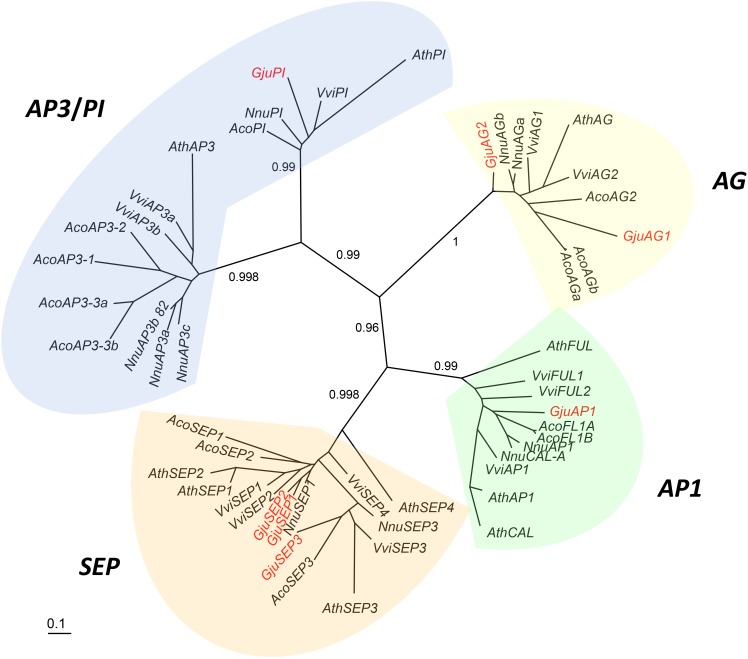
Unrooted phylogram of A-, B-, C-, E-class MADS-box genes. Seven *G. juniperina* full-length transcripts were included in this analysis. Branches with less than 0.5 aLRT values were collapsed. “Gju” is the abbreviation for *Grevillea juniperina*, “Vvi” for *Vitis vinifera*, “Nnu” for *Nelumbo nucifera*, “Ath” for *Arabidopsis thaliana* and “Aco” for *Aquilegia caerulea* genes. Protein IDs are specified in Supplementary Table S2.

A phylogenetic tree including full length *G. juniperina* coding sequences and A-, B-, C-, and E-class MADS-box CDS from *Vitis vinifera*, *Nelumbo nucifera*, *Arabidopsis thaliana*, and *Aquilegia caerulea* was reconstructed using PhyML (Figure 6). We retrieved the A-, B-, C-, and E-class gene lineages with good support, and at least one *G. juniperina* sequence falls in each of these clades. Two *AG*-like *G. juniperina* sequences were found in the C lineage, one of which is closely related to the *N. nucifera* sequences. Three *G. juniperina* sequences were found in the E lineage. One of these fell in the SEP3 clade; the other two grouped together and might correspond to closely related paralogs. Surprisingly in the B-class, no *G. juniperina* sequence was found in the AP3 lineage, while one sequence was found in the PI one, closely related to the two other basal eudicot sequences.

#### Homologs of Genes Co-expressed With ABCE MADS-Box Genes

**FIGURE 7:**
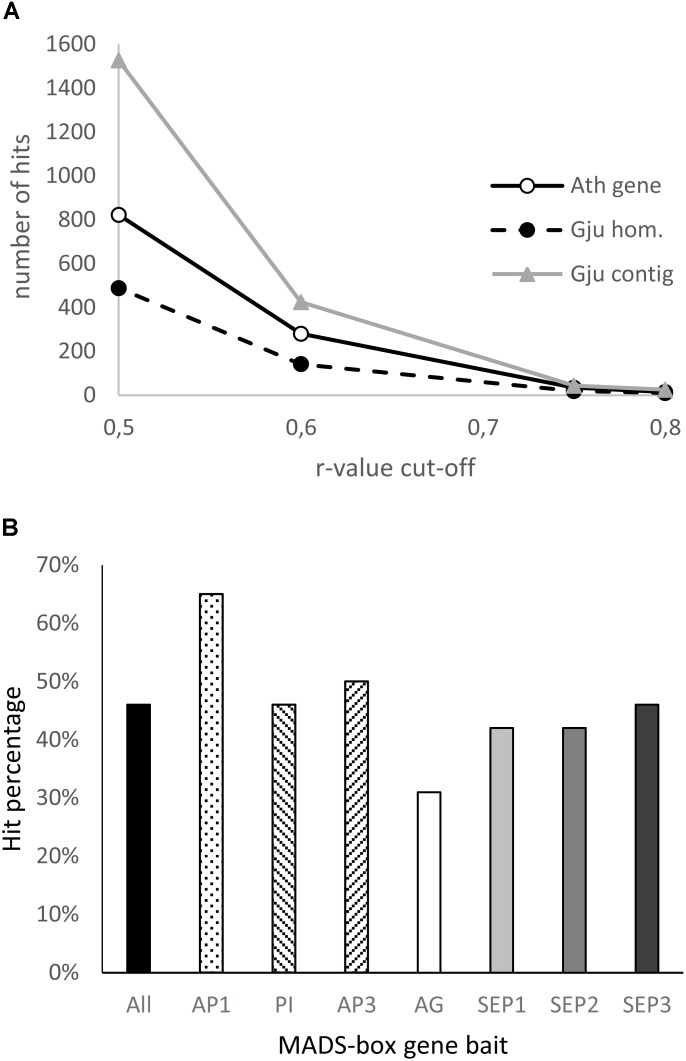
Analysis of the *Grevillea juniperina* homologs of genes co-expressed with the ABCE model MADS-box genes in *Arabidopsis thaliana*. **(A)** Number of *A. thaliana* genes (At gene) of the GRN and their homologous sequences in *G. juniperina* (Gju hom) plotted relative to the Pearson correlation coefficient (*r*-value) cut-off. The number of corresponding contigs for each homolog (Gju contig) are also plotted. **(B)** Percentage of the 25 *A. thaliana* MADS-box best-correlated genes having at least one homolog in *G. juniperina*. The list of the 25 best correlated genes was done using each of the seven *A. thaliana* MADS-box genes as a bait, or a compilation of the best correlated to all the ABCE MADS-box genes (all).

To investigate the extent to which the gene network involved in floral organ development is conserved between *Arabidopsis* and *Grevillea*, we searched for genes with expression patterns that are correlated with that of ABCE MADS-box genes in *Arabidopsis thaliana* using the Expression Angler Website, and then searched for their homologs in the annotated *G. juniperina* transcriptome. In *Arabidopsis*, 822 and 34 genes were found with a Pearson correlation coefficient cut-off (*r*-value) of, respectively, 0.5 and 0.75 (Supplementary Table S3). Analysis of the annotated *G. juniperina* transcriptome indicates global conservation of the gene network between the two species (Figure 7A) although with some variations (Table 1). Homologs of ABCE genes and of their highest correlated genes were found in the *G. juniperina* transcriptome, suggesting they are conserved between the two species (Figure 7A and Table 1). Indeed, 10 out of the 14 *Arabidopsis* genes with an *r*-value of 0.8 have at least one homologous contig in the *G. juniperina* transcriptome.

**Table 1:** List of genes co-expressed with the ABCE MADS-box genes in *Arabidopsis thaliana* (*r*-value ≥ 0.75, in bold the 14 AGI-ID with an *r*-value ≥ 0.8; AGI ID, Arabidopsis Genome Initiative, gene identifier).

AGI ID	Annotation	Gju. Contig number
**With at least one homologous sequence in the *G. juniperina* transcriptome.**		
***At1g24260***	AGL9_SEP3__K-box region and MADS-box transcription factor family protein	2
***At3g02310***	AGL4_SEP2__K-box region and MADS-box transcription factor family protein	4
***At4g18960***	AG__K-box region and MADS-box transcription factor family protein	1
***At5g20240***	PI__K-box region and MADS-box transcription factor family protein	6
***At1g69120***	AGL7_AP1_AtAP1__K-box region and MADS-box transcription factor family protein	1
***At1g78960***	ATLUP2_LUP2__lupeol synthase 2	6
***At1g59640***	BPE_BPEp_BPEub_ZCW32__BIG PETAL P	2
***At1g02190***	Fattyacid hydroxylase superfamily	1
***At3g03450***	RGL2__RGA-like 2	1
***At1g05490***	chr31__chromatin remodeling 31	1
*At2g38110*	ATGPAT6_GPAT6__glycerol-3-phosphate acyltransferase 6	4
*At5g02190*	ATASP38_EMB24_PCS1__Eukaryotic aspartyl protease family protein	1
*At3g01980*	NAD(P)-binding Rossmann-fold superfamily protein	1
*At5g59120*	ATSBT4.13_SBT4.13__subtilase 4.13	1
*At5g35670*	iqd33__IQ-domain 33	1
*At5g33370*	GDSL-like Lipase/Acylhydrolase superfamily protein	5
*At2g20870*	Cell wall protein precursor, putative	1
*At5g22430*	Pollen Ole e 1 allergen and extensin family protein	3
*At4g09960*	AGL11_STK__K-box region and MADS-box transcription factor family protein	1
**With no homologous sequence in the *G. juniperina* transcriptome.**		
**At3g54340**	AP3_ATAP3__K-box region and MADS-box transcription factor family protein	
***At5g15800***	AGL2_SEP1__K-box region and MADS-box transcription factor family protein	
***At4g01080***	TBL26__TRICHOME BIREFRINGENCE-LIKE 26	
***At3g10570***	CYP77A6__cytochrome P450, family 77, subfamily A, polypeptide 6	
*At1g16705*	p300/CBP acetyltransferase-related protein-related	
*At3g04960*	Domain of unknown function (DUF3444)	
*At1g66350*	RGL_RGL1__RGA-like 1	
*At4g21590*	ENDO3__endonuclease 3	
*At1g27360*	SPL11__squamosa promoter-like 11	
*At4g14695*	Uncharacterised protein family (UPF0041)	
*At3g28500*	60S acidic ribosomal protein family	
*At5g48880*	KAT5_PKT1_PKT2__peroxisomal 3-keto-acyl-CoA thiolase 2	
*At1g35170*	TRAM, LAG1, and CLN8 (TLC) lipid-sensing domain containing protein	
*At2g42830*	AGL5_SHP2__K-box region and MADS-box transcription factor family protein	
*At1g72260*	THI2.1_THI2.1.1__thionin 2.1	


Analyses of the 25 best correlated genes to each of the seven *A. thaliana* ABCE MADS-box genes showed an overrepresentation of homologs of the *AP1*-correlated genes and an underrepresentation of the *AG*-correlated genes in the *G. juniperina* annotated transcriptome. The analysis confirmed that no homologs of *AP3* were found in the *G. juniperina* transcriptome (Table 1). Nevertheless, the percentage of *G. juniperina* homologs of *AP3*-correlated genes was similar to the percentage of the homologs of *PI*-correlated genes, or to the percentage computed over all the best ABCE MADS-box correlated genes (Figure 7B).

### Gene Expression During Floral Development

Homologs of genes that play a role in floral meristem termination, organ identity/development and boundaries between organs in *Arabidopsis thaliana* were identified in the *G. juniperina* transcriptome annotated against the *Arabidopsis* proteome. In particular, genes expressed in carpel and/or ovule development in *A. thaliana* (Arnaud and Pautot, 2014; Maugarny et al., 2016) and in other distantly related taxa (Fourquin et al., 2005; Reymond et al., 2012) were targeted, since the gynoecium can easily be separated from perianth with adnate stamens tissues, and its growth pattern is a major component of floral symmetry. In addition, the two previously characterized *ProtCYC1* and *ProtCYC2* genes (Citerne et al., 2017) respectively, *GjuCYC1* and *GjuCYC2*, were included in the expression analysis.

**FIGURE 8:**
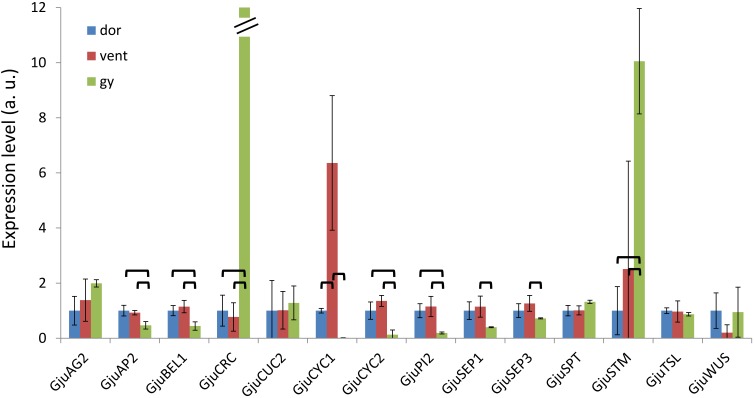
Expression patterns of 14 floral genes assessed by qRT-PCR in floral dissections of *Grevillea juniperina*. Organs were dissected from 6–7 mm buds; dor, dorsal tepals and adnate stamens; vent, ventral tepals and adnate stamens; gy, gynoecium. For a better display, the normalized expression levels of each gene in this figure are shown relative to the dor sample. In this figure, the expression level of *GjuCRC* in the gynoecium is 844. The error bars are calculated from three biological replicates. The statistically significant pairwise comparisons are indicated with brackets.

The expression pattern of these genes was examined in dissected organs from 6 to 7 mm buds using qRT-PCR. *GjuCRC* and *GjuSTM* had a predominant expression in the gynoecium compared to the dorsal and ventral organs, while *GjuAP2*, *GjuBEL1*, *GjuCYC2* and *GjuPI2* were predominantly expressed in the dorsal and ventral organs. *GjuSEP1* and *GjuSEP3* were significantly induced in the ventral organs compared to the gynoecium. Finally, *GjuCYC1* was predominantly expressed in the ventral organs (Figure 8).

## Discussion

By describing specific developmental features of flowers within Proteaceae, we found that the adnation of stamens to tepals takes place at early developmental stages, and that the establishment of bilateral symmetry coincides with asymmetrical growth of the single carpel. The transcriptome data obtained in parallel provides a wide array of genes expressed during floral development, opening the way for future studies to look at the expression of selected candidates within specific organs and/or at specific developmental stages, as discussed below.

### Floral Development

In angiosperms, the initiation of floral organs typically follows a highly conserved pattern, with a centripetal initiation of the organs from the outermost to the innermost (generally the gynoecium) (Endress, 2006). However, there is no general rule as to when symmetry is established during development (Endress, 1999). In species with zygomorphic flowers, bilateral symmetry can be visible very early on during development; alternatively, floral buds can remain actinomorphic until late stages of development. Furthermore, species with actinomorphic flowers at anthesis may undergo transitory bilateral symmetry of the floral bud during development (reviewed in Reyes et al., 2016). At the gynoecium level, the type of symmetry is conditioned by the number of carpels and their closure pattern, which can be either ascidiate or plicate (Endress, 2015; Sokoloff et al., 2017). In species with a single plicate carpel presenting a marked cleft during development, as it is the case in Proteaceae (Douglas and Tucker, 1996b), the gynoecium becomes zygomorphic as soon as the cleft begins to form. In most subfamilies of Proteaceae, the cleft is typically oriented along the dorso-ventral axis, facing the adaxial tepal. In Grevilloideae, however, the situation is more complex, as described in detail by Douglas and Tucker (1996b) (see also Johnson and Briggs, 1975), who suggested that the variation in carpel orientation could be related to the space left after stamen initiation. Our observations suggest asymmetrical growth of the carpel primordium from the very early stages, resulting in early zygomorphy of the gynoecium. The orientation of flowers within the flower pair is also variable (Douglas and Tucker, 1996a), making the interpretation of axes of symmetry even more complex. Such variation in carpel and flower orientation has been suggested to be related to the inflorescence structure and degree of compaction that are both variable across the family (Johnson and Briggs, 1975). In Embothrieae, the tribe to which our four study species belong, zygomorphy is widespread and has been inferred as the ancestral condition (Citerne et al., 2017). The different degrees of floral bilateral symmetry at anthesis in the species examined here could be related to differences in inflorescence morphology. How the shape and compaction of the inflorescence may condition flower symmetry remains to be explored. The fact that *ProtCYC* genes are expressed (although faintly in the case of *ProtCYC1*) in the gynoecium of *G. juniperina* (Citerne et al., 2017 and this study), and asymmetrically in the tepals and/or adnate stamens (*ProtCYC1* only), suggests that these genes could play a role in the genetic control of floral zygomorphy in Proteaceae, as already suggested by Citerne et al. (2017).

The position of stamens, opposite the tepals, questions the identity of the perianth. In whorled flowers, the stamens usually alternate with adjacent perianth parts when in equal number. In the order Ericales, obhaplostemony (petal-opposed stamens) is believed to be derived from diplostemony (Schönenberger et al., 2005), with the loss of a stamen whorl. Detailed expression studies of the homologs of floral organ identity genes and their correlated genes found in the *G. juniperina* transcriptome may provide clues as to the homology of the perianth. Furthermore, the transcriptomic data may also help to investigate the genetic control of organ fusion.

### A Transcriptomic Tool to Investigate the Genetic Bases of Floral Development

Transcriptomic data from Proteaceae species are scarce and have mostly been obtained from leaves for specific purposes: in *Leucadendron* to derive phylogenetic markers (Tonnabel et al., 2014), in *Protea repens* to study population differentiation in relation to local adaptation (Akman et al., 2016), in *Banksia hookeriana* to derive SSR markers (Lim et al., 2017) and in *Gevuina avellana* to study heteroblastic development under different light conditions (Ostria-Gallardo et al., 2016). In *Macadamia integrifolia*, a reference transcriptome was built from flowers, shoots and leaves, in parallel to a draft genome (Nock et al., 2016). Our *Grevillea juniperina* transcriptome was built from an equal mix of RNAs from young leaves and flower buds at three developmental stages, all posterior to organ inception. The general metrics of this transcriptome such as N50, mean length of contigs, percentage of annotated contigs, all fall within the range of values obtained in other Proteaceae species (e.g., Akman et al., 2016). The sequencing effort and mix of tissues were designed to cover a large panel of expressed genes including transcription factors, with a higher proportion of floral samples in the library used for RNA-seq to obtain a good coverage of transcripts expressed during floral development. We probably missed some rare floral transcripts, such as the *ProtCYC2* gene that we previously found to be expressed in floral buds of the same species of *Grevillea* (Citerne et al., 2017), and whose expression was confirmed here. Equally, the absence of a homolog of the B-class gene *AP3* is intriguing, because we were able to find at least one homologous contig for all the other A-, B-, C-, and E-class genes. The fact that we found *G. juniperina* homologs of *A. thaliana* genes with correlated expression with *AP3* would rather be in favor of a technical bias. Gene expression analysis showed expected patterns for the homologs of the identity genes tested, including *GjuAP2*, a homolog of the *A. thaliana* *AP2* gene, which is one of the two A-class genes and the only ABCE model gene that is not from the MADS-box gene family. TCP genes are also well represented in the transcriptome, with 14 and seven homologs belonging to class I and class II subfamilies, respectively, which is comparable to the size of the family in many eudicots. One sequence matched the previously characterized *ProtCYC1* gene, which is homologous to the *Arabidopsis* *CYC*-like genes (*TCP18/TCP12/TCP1*). This gene is the only one exhibiting an asymmetric expression pattern in the floral dissection that could be related to the asymmetry of the perianth and stamens, as already observed by Citerne et al. (2017) in this species and another zygomorphic *Grevillea* species.

ABCE MADS-box genes are master regulators of the floral organ development Gene Regulatory Network (GRN) that are subjected to strong functional constraints, in contrast with the great flexibility of organ morphogenesis (Davila-Velderrain et al., 2013; Becker and Ehlers, 2016). Genes co-expressed with these regulatory genes in *Arabidopsis* could serve as baits to identify conserved modules of connected genes in other species and analyze their evolution and expression patterns. A large proportion of genes with expression patterns that are highly correlated with floral organ identity gene expression in *Arabidopsis thaliana* have homologs in the *G. juniperina* transcriptome (10 out of 14). Available functional studies in *A. thaliana* indicate that these genes code for transcription factors and/or hormonal signaling pathway proteins, and are important regulators of flower organ development, sexual reproduction, and seed fertility (Ge et al., 2005; Szecsi et al., 2006; Hou et al., 2008; Varaud et al., 2011; Li et al., 2012; Hong et al., 2017). A less stringent correlation threshold gives a lesser proportion of homologous genes in the *G. juniperina* transcriptome. This may suggest that beyond a core set of genes whose expression patterns are strongly parallel to that of organ identity genes, other genes involved in flower development have diverged in the two species. It is worth noting the relative overrepresentation in *G. juniperina* of homologs of *AP1*-correlated genes. It remains to be seen whether this finding may be relevant for assessing the homology of undifferentiated perianth organs with sepals or petals. Among the genes expressed during carpel and ovule development in *Arabidopsis*, only *GjuCRC* and *GjuSTM* exhibit a predominant expression in the gynoecium of *G. juniperina*, while *GjuAG2* was also found to be expressed in perianth and stamen tissues (both ventral and dorsal organs). Detailed expression analysis of these genes at the tissue level (using *in situ* hybridization for example) may provide insight into the processes involved in the monosymmetric growth of the gynoecium during floral ontogeny.

Floral evo–devo questions that concern Proteaceae are many, among which the homology of the undifferentiated perianth to a calyx or a corolla, the adnation of stamens, or the chronology and developmental processes of the acquisition of zygomorphy. Such issues could be addressed by combining developmental and molecular approaches, for example using RNA-seq at different floral developmental stages in a panel of well-chosen species. The *G. juniperina* reference transcriptome will be a valuable resource for all these future studies. It will be a reference for identifying differentially expressed genes related to the establishment of symmetry at appropriate developmental stages in different floral organs, and for investigating the gene network underlying tepal development, providing insights into the homology of the colored perianth organs. Choosing appropriate developmental stages may be facilitated by High Resolution X-Ray Computed Tomography giving access to early developmental stages without dissection, which is especially valuable for species with young compact and tough inflorescences.

## Author Contributions

SN and CD designed the project. HS, FS, YS, JS, and SN performed the developmental analysis. ED and JY generated RNA-seq data and transcriptome assembly. JJ, NCS, YD, HC, CD, and FS annotated and mined transcriptomic data. ED and MLG performed the expression analysis. All authors contributed to the manuscript.

## Conflict of Interest Statement

The authors declare that the research was conducted in the absence of any commercial or financial relationships that could be construed as a potential conflict of interest.

## Footnotes

1 http://www.environment.gov.au/science/abrs/online-resources/flora-of-australia-online

2 http://www.bioinfogenome.net/OrthoVenn/

3 https://www.ebi.ac.uk/Tools/msa/muscle/

4 https://www.arabidopsis.org/

5 https://www.ncbi.nlm.nih.gov/

6 https://phytozome.jgi.doe.gov/

7 http://translatorx.co.uk/
