# Supplementary material for: Unraveling the Developmental and Genetic Mechanisms Underpinning Floral Architecture in Proteaceae
Source: Front Plant Sci. 2019 Jan 25;10:18. doi: 10.3389/fpls.2019.00018 (PMC6357683; doi:10.3389/fpls.2019.00018)
Supplement: Supplementary file 1 [file Table_1.DOCX]

Supplementary Table S1 - Scanning parameters for High Resolution X-Ray Computed Tomography

| Species | Figure  number | source  power [W] | acceleration  voltage [kV] | exposure  time [s] | camera  binning | pictures  per sample | optical  magnification | Voxel  size [mm] |
| --- | --- | --- | --- | --- | --- | --- | --- | --- |
| *Grevillea juniperina* | 2C | 2,25 | 25 | 20 | 1 | 1000 | 10x | 1,05 |
| *Grevillea juniperina* | 2D | 2,275 | 25 | 30 | 1 | 1000 | 20x | 0.54 |
| *Stenocarpus davallioides* | 3A | 2,28 | 25 | 20 | 1 | 728 | 10x | 1,03 |
| *Stenocarpus davallioides* | 3B | 2,28 | 25 | 20 | 1 | 728 | 10x | 1,03 |
| *Stenocarpus davallioides* | 3C | 2,25 | 25 | 10 | 1 | 1200 | 4x | 2,48 |
| *Alloxylon flammeum* | 3D | 2,30 | 25 | 1 | 2 | 728 | 4x | 5,06 |
| *Alloxylon flammeum* | 3E | 2,275 | 25 | 18 | 1 | 728 | 10x | 0.97 |
| *Grevillea petrophiloides* | 3F | 1,75 | 50 | 5 | 1 | 728 | 4x | 2.56 |
|  |  |  |  |  |  |  |  |  |
