# Supplementary material for: Unraveling the Developmental and Genetic Mechanisms Underpinning Floral Architecture in Proteaceae
Source: Front Plant Sci. 2019 Jan 25;10:18. doi: 10.3389/fpls.2019.00018 (PMC6357683; doi:10.3389/fpls.2019.00018)
Supplement: Supplementary file 6 [file Data_Sheet_1.PDF]

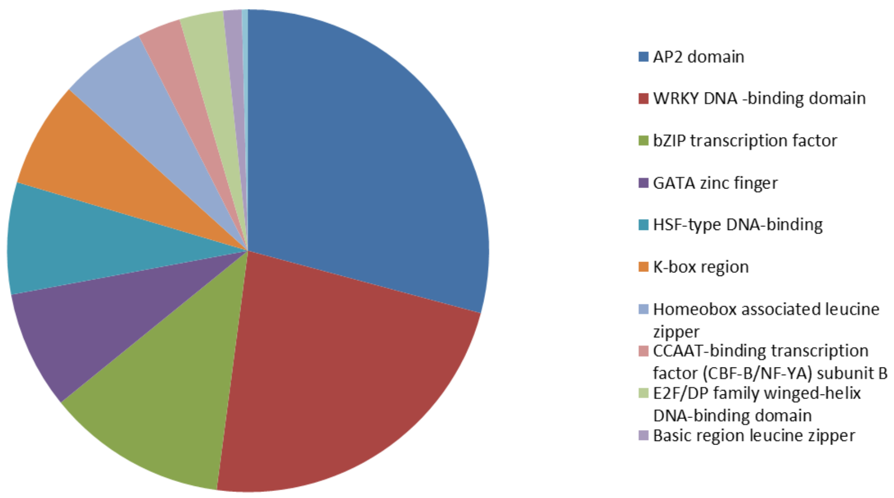

Supplementary Figure 1. Pie chart of the transcription factor domains identified in 240 contigs annotated in the GO: 0003700 in InterProScan.

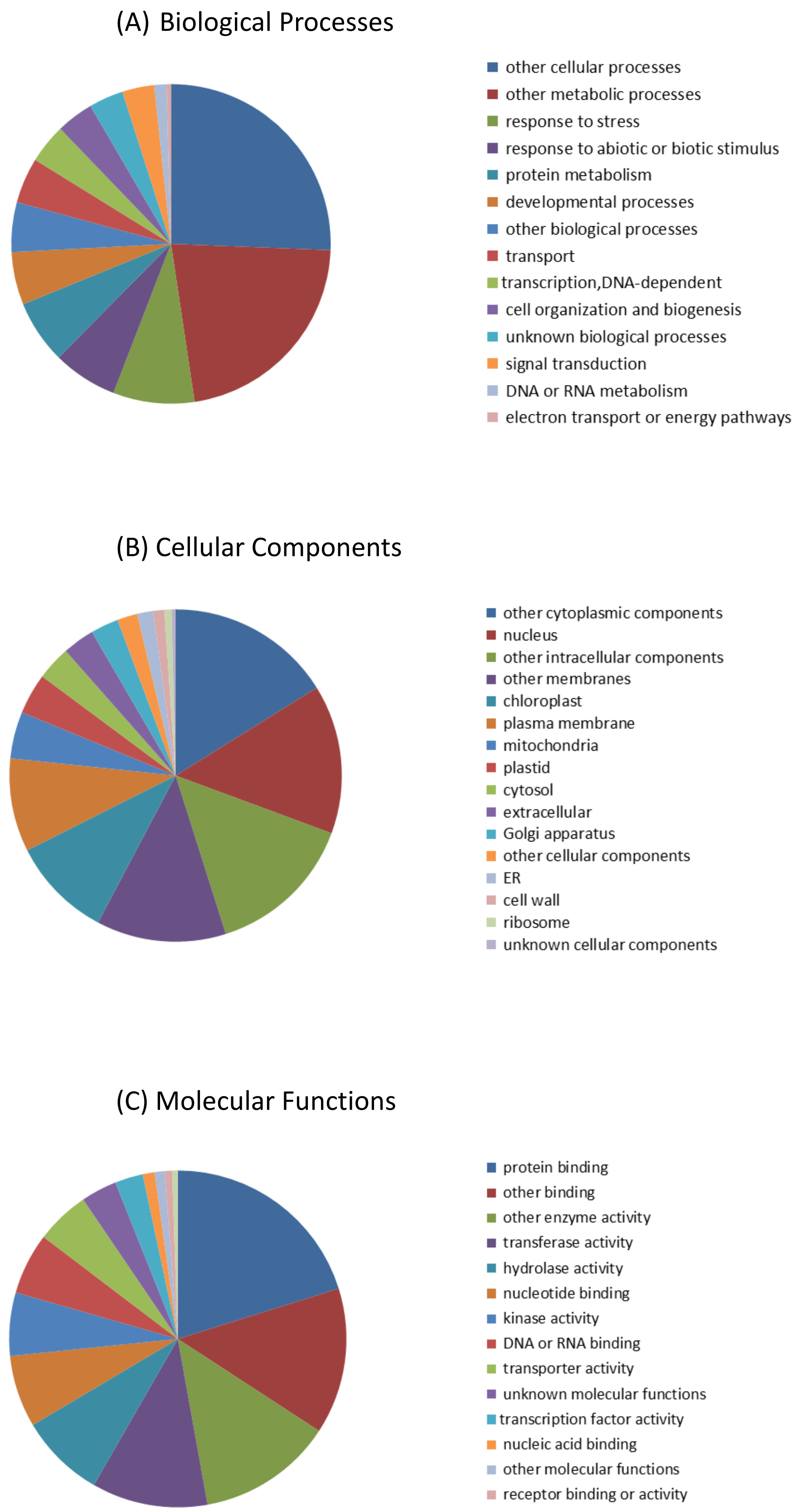

Supplementary Figure 2. Pie charts of GO Slim functional annotations for Biological process, Cellular Component and Molecular function categories.

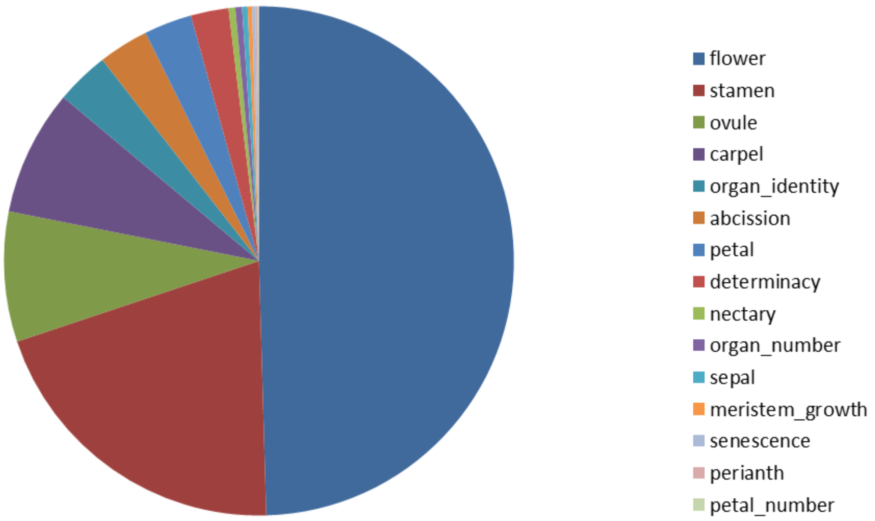

Supplementary Figure 3. Distribution of functional annotations corresponding to GO term GO:0009908 and all its children terms in floral organs or developmental processes.
